# Cultural and linguistic adaption and testing of the Health Literacy Questionnaire (HLQ) among healthy people in Korea

**DOI:** 10.1371/journal.pone.0271549

**Published:** 2022-08-01

**Authors:** Jin-Hee Park, Richard H. Osborne, Hee-Jun Kim, Sun Hyoung Bae

**Affiliations:** 1 College of Nursing Research Institute of Nursing Science, Ajou University, Suwon, Korea; 2 Centre for Global Health and Equity, School of Health Sciences, Swinburne University of Technology, Melbourne, Australia; Post Graduate Institute of Medical Education and Research, INDIA

## Abstract

**Backgrounds:**

This study administered the Health Literacy Questionnaire (HLQ) among Korean adults to examine its factor structure, reliability, and validity.

**Methods:**

The HLQ items were translated and culturally adapted to the Korean context. The convenience sampling method was used, and data were collected. The difficulty level, confirmatory factor analysis (CFA) using diagonal weighted least squares (DWLS) estimator in R, discriminant validity, and composite reliability were performed.

**Results:**

The easiest scale to obtain a high score was “Scale 4. Social support for health” and the hardest was “Scale 7. Navigating the healthcare system.” Nine one-factor models fitted well. The nine-factor structural equation model fitted the data well. All HLQ scales were homogenous, with composite reliability.

**Conclusions:**

The Korean version of the HLQ has a strong construct and high composite reliability when applied to Korean adults.

## Introduction

Health literacy refers to the ability to understand health information that is essential to managing diseases, promoting health, and engaging in action. It encompasses comprehension of various medical information, including diet, exercise, prescriptions, and medication instructions. According to the US National Institute of Health, health literacy is the “degree to which individuals have the ability to find, understand, and use information and services to make health-related decisions and actions” [1]. The World Health Organization defines health literacy as “the cognitive and social skills which determine the ability of individuals to understand and use information in ways that promote and maintain good health” [2]. In essence, health literacy is an individual’s ability to acquire, process, and understand health information and services to make informed health-related decisions [3].

People with poor health literacy have difficulty understanding information related to health, show poor medication adherence and self-care [4,5], and do not practice preventive behaviors, such as cancer screening [6]. Furthermore, they experience difficulty in obtaining and understanding basic health information and making appropriate decisions, leading to adverse health effects, such as increased smoking and rate of hospitalization [7,8]. Health literacy impacts health and diseases; therefore, improving health literacy is crucial [8]. Thus, individuals’ health literacy should be first assessed to provide tailored health information and education, and a valid instrument is required to conduct an accurate health literacy assessment [9,10].

Early instruments used to measure health literacy include the Rapid Estimate of Adult Literacy in Medicine (REALM), Test of Functional Health Literacy in Adults and the Newest Vital Sign. The Test of Functional Health Literacy in Adults is a 67-item tool used only for assessing comprehension and numeracy. One disadvantage of this instrument is that it has no time limitation to complete the test; therefore, the time taken to perform the test was rarely reported [11]. The REALM includes questions regarding medical terminology [12], and in Korea, the Korean Health Literacy Assessment Tool adapted by Kim et al. [13] is used. However, this 66-item tool only assesses comprehension of medical terminology. The Newest Vital Sign is a simple six-item instrument that contains questions concerning calculations of calories and percentage of nutrients using a picture of ice cream. However, one of its shortcomings is that health literacy is determined based on mathematical skills [14]. None of these instruments measure the multidimensional nature of health literacy as described by accepted definitions of the construct.

Health literacy is beyond reading and comprehending health information and encompasses the ability to approach, comprehend, judge, and use information [9]. Thus, instruments used to measure health literacy must assess these wide-ranging factors. Osborne et al. [15] developed the Health Literacy Questionnaire (HLQ); it is used to identify health literacy strengths, needs and preferences of individuals and groups in communities, and clinical practices. In recent years, the HLQ has become one of the most frequently utilized health literacy instruments in public health and health services research. The HLQ comprises nine subscales that capture the overall health literacy of individuals and groups and has been translated into over 30 languages, including European languages (Dutch [16], Danish [17], German [18], French [19]), and Slovakian [20], as well as African [21,22]. Health literacy involves solving health-related problems within the contexts of the societies in which people live [2]. Therefore, measurement of health literacy using an instrument developed in one cultural context involves testing the extent to which data derived from the instrument are valid in another cultural context, particularly within the healthcare environment of that country or society [23]. The aim of this study was to translate the HLQ into Korean and conduct psychometric analyses (item difficulty, confirmatory factor analysis, reliability) on data derived from the Korean version of the HLQ administered to adults living in Seoul, Korea.

## Materials and method

### Design and sample

This is a methodological study to test the validity and reliability of the Korean version of the HLQ for measuring health literacy. The convenience sampling method was used in this study. The participants were randomly recruited online via “Google^®^ Forms” by announcing recruitment on a hospital bulletin board for participants who met the inclusion criteria in the metropolitan area. The inclusion criteria were (a) adults 19 years and over and (b) ability to speak and understand Korean. Confirmatory factor analysis (CFA) requires a minimum of 300 participants [24]; we enrolled 450 participants due to potential dropouts. A total of 450 people participated in the survey, and after excluding 31 who dropped out during the survey, 419 people were included in the final analysis.

### Instruments

#### The Health Literacy Questionnaire

The HLQ comprises 44 items representing nine independent health literacy scales [15]. The nine scales are 1. Feeling understood and supported by healthcare providers (4 items), 2. Having sufficient information to manage my health (4 items), 3. Actively managing my health (5 items), 4. Social support for health (5 items), 5. Appraisal of health information (5 items), 6. Ability to actively engage with healthcare providers (5 items), 7. Navigating the healthcare system (6 items), 8. Ability to find good health information (5 items), 9. Understand health information well enough to know what to do (5 items). Scales 1–5 (Part 1) are scored using response options indicating the level of agreement (ranging from 1 = strongly disagree to 4 = strongly agree). Scales 6–9 (Part 2) are scored using response options indicating difficulty (from 1 = cannot do or usually difficult to 5 = very easy). The results are nine scale scores, which were calculated as the average of the item scores of each scale, with higher scores indicating potential health literacy strengths.

### Translation of the Health Literacy Questionnaire (HLQ)

Following consultation with the original authors of the HLQ, a license to adapt the HLQ was obtained (#TL1806IA 12/1/2018). Translation and back-translation were performed in accordance with the Swinburne Translation Integrity Procedure [25]. This is a systematic translation method that uses item intents (descriptions of the meanings of items) to support forward translators to choose words and phrases that maximize measurement equivalence across languages and also considers linguistic and cultural appropriateness [25,26]. In step one, two Korean native translators independently translated the English version into Korean (forward translation). In step two, a third Korean native reviewed the two translations to choose the best one, combine the two, or propose another translation (Consensus forward translation). In step three, one bilingual translator fluent in Korean and English back-translated the Korean-translated version into English (Back-translation). The bilingual translator had expert knowledge in medicine and public health and did not participate in the translations in steps one and two. In step four, the questionnaire developer, the Korean translation team, and two bilinguals conducted a quality assurance consensus meeting via teleconference. During this meeting, each translated item was reviewed to assess cultural appropriateness and measurement invariance. Most of the forward translations were accepted during the consensus discussion. However, some items were revised through discussion to better reflect the intended meaning of the item. For example, “I feel I have good information about…” (Scale 2) was revised to “I feel there is good information available to me about…” In step five, the completed translation was edited for accurate grammar and spelling to finalize the Korean version of the HLQ.

### Data collection

The Institutional Review Board of Ajou University Hospital (AJIRB-SBR-SUR-21-117), the authors’ affiliated university hospital, reviewed and approved research procedures. Thereafter, data were collected through Google^®^ Forms from April 28 to September 11, 2021. The survey link was published on a medical center dashboard. Information about the study was also posted on institutional homepages for recruitment. The study announcement described the study’s purpose, method, and selection criteria. We provided a Quick Response code to access detailed information regarding the study and survey. Potential participants accessed the survey link using the Quick Response code once they agreed to participate. Due to the nature of the online survey, voluntary participation was considered as consent to enroll in the study. Study participants received mobile gift cards ($5) after they completed the survey. A detailed explanation of the research was provided in a separate explanatory note, including a clause that there would be no disadvantages due to participation or non-participation in the research. The survey took approximately 15 minutes to complete. The study was conducted according to the Declaration of Helsinki principles. A formal informed consent was waived due to the nature of the online survey.

### Statistical analysis

IBM SPSS Statistics 26.0, R 4.1.2 software was used to analyze data. Descriptive statistics was used to analyze participants’ demographic characteristics. The mean score, response range of each item, and the estimated difficulty of the nine scales were analyzed. The HLQ was completed in two ways. Therefore the difficulty was also assessed using two methods. First, Part 1 (Scales 1–5) was rated as agree/disagree. The difficulty was determined based on the percentage of “strongly disagree” or “disagree” against “agree” or “strongly agree.” Thereafter, Part 2 (Scales 6–9) was rated based on the percentage of “cannot do or always difficult,” “usually difficult,” and “sometimes difficult” against “usually easy” and “always easy” [15].

The construct validity of the HLQ has been established in various languages [15,19,27]; therefore, the construct validity of the Korean version of the HLQ was evaluated using CFA. The items of the HLQ were rated on four and five-point ordinal responses. Thus, ordinal variables were analyzed with diagonal weighted least squares (DWLS) [28]. The latent variable analysis package of the R software was used [29]. First, a one-factor CFA model was conducted to determine whether the data for each factor fitted the model. The standardized and non-standardized factor loadings, R^2^ (variance of observed variable explained by latent variables), standard error, and 95% confidence interval (CI) of the observed variables were analyzed. One-factor CFA model fit was tested using chi-square, Comparative Fit Index (CFI), Tucker–Lewis Index (TLI), Root Mean Square Error of Approximation (RMSEA), and the Standard Root Mean Square Residual (SRMR). A CFI > 0.95, TLI > 0.95, SRMR< 0.08 and RMSEA< 0.06 indicated a close fit, and 0.06 < RMSEA< 0.08 indicated a reasonable fit. Thereafter, a strict nine-factor CFA model without correlated residuals or cross-loadings was fitted to the data. Discriminant validity was assessed by analyzing inter-factor correlations with Spearman rank correlation, and a correlation coefficient of 0.8 or higher indicated a low discriminant validity.

Reliability was evaluated using Cronbach’s α and composite reliability (CR) for internal consistency [30]. Cronbach’s α is a biased estimate of reliability for consistency; items are correlated or loaded onto the same factor [31]. Thus, CR provides an unbiased reliability estimate of the study population [15,30]. In this study, we calculated both Cronbach’s α and CR with reference to the literature [15,18,19,27]. A CR of .70 or higher and .95 or lower was deemed desirable [32].

## Results

### Sociodemographic characteristics of participants

The sociodemographic characteristics of participants are shown in Table 1. The total participants consisted of 68.7% women from 20 to 82 years, with a mean age of 48.8 years (SD = 13.7). Approximately 7.2% of the participants had under middle school level education, which is compulsory education in Korea. Over 70% of participants reported high economic levels, and less than 10% lived alone. Approximately 50% of the participants were diagnosed with at least one chronic disease, such as hypertension, diabetes, or arthritis, and were receiving treatment, and 71.8% reported high interest in their health (Table 1).

**Table 1:** Sociodemographic characteristics of Participants (N = 419).

Characteristics	Categories	n (%)	Mean ± SD
Gender	Male	131 (31.3)	
	Female	288 (68.7)	
Age (yr, range: 20–82)	< 34	70 (16.7)	48.8 **±** 13.7
	35~44	100 (23.9)	
	45~54	91 (21.7)	
	55~64	93 (22.2)	
	≥65	65 (15.5)	
Educational level	Middle school or lower	30 (7.2)	
	High School	150 (35.8)	
	College or above	239 (57.0)	
Perceived economic status	Low	5 (1.2)	
	Middle	91 (21.7)	
	High	323 (77.1)	
Living alone	Yes	40 (9.5)	
	No	379 (90.5)	
Chronic disease	Yes	194 (46.3)	
	No	225 (53.7)	
Perceived the level of interest in health	Low	118 (28.2)	
	High	301 (71.8)	

### The difficulty level of the Korean version of the HLQ

The level of difficulty of the Korean version of the HLQ ranged from 11.6–46.7% for Part 1. Scale 4. Social support for health was the least difficult (11.6%); however, Scale 2. Having sufficient information to manage my health was the most difficult (46.7%). The difficulty of Part 2 ranged from 53.1–55.7% and was higher than that for Part 1. Scale 8. Ability to find good health information was the least difficult (53.1%), and Scale 7. Navigating the healthcare system was the most difficult (55.7%) (Table 2).

**Table 2:** Difficulty level of the items and psychometric properties of HLQ using one-factor and nine-factor CFA model (n = 419).

Scales / Items ^a^	Mean ± SD	Scale’s Mean ± SD	Difficulty level % (95% CI) ^b^	Average item difficulty (%)	One-factor CFA		Nine-factor CFA	
					Factor loadings	R^2^	Factor loadings	R^2^
**Part 1: Scales 1–5: how strongly you disagree or agree with the following statements (strongly disagree/disagree/agree/strongly agree)**								
**Scale 1. Feeling understood and supported by healthcare providers (HPS)**								
1. I have at least one healthcare provider…	2.55 ± 0.77	2.65 ± 0.60	40.6 (35.9–45.3)	36.2	0.892	0.796	0.820	0.673
2. I have at least one healthcare provider…	2.74 ± 0.66		29.4 (25.0–33.7)		0.888	0.789	0.867	0.752
3. I have the healthcare providers I need to help me…	2.59 ± 0.68		43.7 (38.9–48.4)		0.835	0.697	0.935	0.875
4. I can rely on at least one…	2.71 ± 0.67		31.0 (26.6–35.5)		0.857	0.735	0.854	0.729
**Model fit (one-factor CFA)—χ**^**2**^ **DWLS (2) = 36.164, *p* < 0.001, CFI = 0.993, TLI = 0.980, RMSEA = 0.202 and SRMR = 0.018** **Cronbach’s a = 0.923, Composite reliability = 0.868**								
**Scale 2. Having sufficient information to manage my health (HSI)**								
1. I feel I have good information about …	2.68 ± 0.62	2.54 ± 0.53	33.7 (29.1–38.2)	46.7	0.782	0.611	0.757	0.574
2. I have enough information to help me…	2.58 ± 0.65		44.6 (39.9–49.4)		0.856	0.733	0.866	0.751
3. I am sure I have all the information I need…	2.45 ± 0.64		54.2 (49.4–59.0)		0.854	0.730	0.832	0.693
4. I have all the information I need…	2.44 ± 0.64		54.2 (49.4–59.0)		0.841	0.707	0.873	0.761
**Model fit (one-factor CFA)—χ**^**2**^ **DWLS (2) = 7.511, *p* = 0.023, CFI = 0.998, TLI = 0.995, RMSEA = 0.081 and SRMR = 0.016** **Cronbach’s a = 0.901, Composite reliability = 0.833**								
**Scale 3. Actively managing my health (AMH)**								
1. I spend quite a lot of time actively …	2.50 ± 0.72	2.60 ± 0.57	51.3 (46.5–56.1)	41.6	0.872	0.760	0.884	0.781
2. I make plans for what I need to do …	2.60 ± 0.68		41.5 (36.8–46.3)		0.840	0.706	0.888	0.788
3. Despite other things in my life, I make time to …	2.59 ± 0.66		43.0 (38.2–47.7)		0.917	0.840	0.877	0.769
4. I set my own goals …	2.61 ± 0.67		40.3 (35.6–45.1)		0.866	0.750	0.881	0.776
5. There are things that I do regularly …	2.71 ± 0.66		32.0 (27.5–36.5)		0.856	0.732	0.822	0.676
**Model fit (one-factor CFA)—χ**^**2**^ **DWLS (5) = 49.288, *p* < 0.001, CFI = 0.994, TLI = 0.987, RMSEA = 0.146 and SRMR = 0.027** **Cronbach’s a = 0.939, Composite reliability = 0.891**								
**Scale 4. Social support for health (SS)**								
1. I can get access to several people …	3.01 ± 0.57	3.02 ± 0.41	12.2 (9.0–15.3)	11.6	0.797	0.635	0.767	0.588
2. When I feel ill, the people …	3.02 ± 0.52		10.0 (7.1–12.9)		0.746	0.557	0.787	0.619
3. If I need help, I have plenty of…	2.87 ± 0.62		22.2 (18.2–26.2)		0.888	0.789	0.953	0.908
4. I have at least one person who can come to …	3.16 ± 0.49		3.6 (1.8–5.4)		0.685	0.469	0.570	0.325
5. I have strong support from family …	3.02 ± 0.50		10.0 (7.1–12.9)		0.810	0.656	0.787	0.619
**Model fit (one-factor CFA)—χ**^**2**^ **DWLS (5) = 16.305, *p* = 0.006, CFI = 0.994, TLI = 0.989, RMSEA = 0.074 and SRMR = 0.024** **Cronbach’s a = 0.889, Composite reliability = 0.806**								
**Scale 5. Appraisal of health information (CA)**								
1. I compare health information …	2.76 ± 0.66	2.74 ± 0.47	28.4 (24.1–32.7)	30.4	0.701	0.492	0.700	0.489
2. When I see new information about health, …	2.79 ± 0.62		27.9 (23.6–32.2)		0.849	0.720	0.742	0.551
3. I always compare health information …	2.82 ± 0.62		23.6 (19.5–27.7)		0.892	0.796	0.759	0.577
4. I know how to find out if the health information I receive …	2.67 ± 0.64		36.8 (32.1–41.4)		0.599	0.359	0.840	0.706
5. I ask healthcare providers about …	2.65 ± 0.66		35.3 (30.7–39.9)		0.601	0.361	0.772	0.596
**Model fit (one-factor CFA)—χ**^**2**^ **DWLS (5) = 73.636, *p* < 0.001, CFI = 0.967, TLI = 0.935, RMSEA = 0.181 and SRMR = 0.061** **Cronbach’s a = 0.845, Composite reliability = 0.809**								
**Part 2: Scales 6–9: How easy or difficult the following tasks are for you to do now (cannot do or always difficult/usually difficult/sometimes difficult/usually easy/always easy)**								
**Scale 6. Ability to actively engage with healthcare providers (AE)**								
1. Make sure that healthcare providers …	3.30 ± 0.91	3.27 ± 0.89	56.6 (51.8–61.3)	55.4	0.882	0.779	0.885	0.784
2. Feel able to discuss your health …	3.33 ± 1.00		52.7 (47.9–57.5)		0.884	0.781	0.857	0.735
3. Have good discussions about …	3.35 ± 1.02		49.6 (44.8–54.4)		0.879	0.772	0.874	0.764
4. Discuss things with healthcare …	3.11 ± 1.04		62.8 (58.1–67.4)		0.874	0.763	0.886	0.786
5. Ask healthcare providers questions …	3.24 ± 1.06		55.1 (50.3–59.9)		0.914	0.836	0.929	0.862
**Model fit (one-factor CFA)—χ**^**2**^ **DWLS (5) = 38.810, *p* < 0.001, CFI = 0.996, TLI = 0.993, RMSEA = 0.127 and SRMR = 0.017** **Cronbach’s a = 0.947, Composite reliability = 0.927**								
**Scale 7. Navigating the healthcare system (NHS)**								
1. Find the right …	3.39 ± 0.94	3.29 ± 0.86	52.5 (47.7–57.3)	55.7	0.797	0.635	0.820	0.672
2. Get to see the healthcare providers you …	3.36 ± 0.99		50.6 (45.8–55.4)		0.885	0.783	0.878	0.771
3. Decide which healthcare provider …	3.21 ± 1.01		60.6 (55.9–65.3)		0.914	0.836	0.912	0.832
4. Make sure you find the right place …	3.34 ± 0.98		52.5 (47.7–57.3)		0.888	0.789	0.880	0.774
5. Find out what healthcare services you are ….	3.26 ± 1.02		54.9 (50.1–59.7)		0.924	0.853	0.909	0.826
6. Work out what the best …	3.19 ± 1.01		63.2 (58.6–67.9)		0.837	0.701	0.867	0.753
**Model fit (one-factor CFA)—χ**^**2**^ **DWLS (9) = 49.999, *p* < 0.001, CFI = 0.997, TLI = 0.994, RMSEA = 0.104 and SRMR = 0.017** **Cronbach’s a = 0.950, Composite reliability = 0.933**								
**Scale 8. Ability to find good health information (FHI)**								
1. Find information about …	3.43 ± 0.89	3.34 (0.81)	49.6 (44.8–54.4)	53.1	0.854	0.729	0.830	0.689
2. Find health information from several different …	3.47 ± 0.90		47.3 (42.5–52.1)		0.910	0.827	0.870	0.758
3. Get information about health so you ….	3.31 ± 0.92		55.6 (50.8–60.4)		0.866	0.750	0.893	0.798
4. Get health information in ….	3.26 ± 0.97		56.6 (51.8–61.3)		0.862	0.743	0.884	0.781
5. Get health information …	3.24 ± 1.02		56.6 (51.8–61.3)		0.882	0.778	0.896	0.802
**Model fit (one-factor CFA)—χ**^**2**^ **DWLS (5) = 65.028, *p* < 0.001, CFI = 0.993, TLI = 0.986, RMSEA = 0.169 and SRMR = 0.027** **Cronbach’s a = 0.940, Composite reliability = 0.917**								
**Scale 9. Understand health information well enough to know what to do (UHI)**								
1. Confidently fill medical forms …	3.25 ± 0.99	3.30 ± 0.81	59.2 (54.5–63.9)	54.8	0.798	0.637	0.820	0.673
2. Accurately follow the instructions …	3.47 ± 0.89		47.0 (42.2–51.8)		0.761	0.579	0.768	0.589
3. Read and understand …	3.40 ± 0.97		51.6 (46.7–56.4)		0.881	0.776	0.864	0.747
4. Read and understand all …	2.98 ± 1.11		67.3 (62.8–71.8)		0.783	0.614	0.783	0.613
5. Understand what healthcare providers …	3.40 ± 0.96		48.9 (44.1–53.7)		0.888	0.789	0.884	0.782
**Model fit (one-factor CFA)—χ**^**2**^ **DWLS (5) = 61.312, *p* < 0.001, CFI = 0.987, TLI = 0.975, RMSEA = 0.164 and SRMR = 0.034** **Cronbach’s a = 0.910, Composite reliability = 0.888**								
**Model fit (nine-factor CFA)—χ**^**2**^ **DWLS (866) = 1993.353, *p*< 0.001, CFI = 0.975, TLI = 0.973, RMSEA = 0.056 and SRMR = 0.054**								

CFA, confirmatory factor analysis; CFI, comparative fit index; CI, confidence interval; df, degree of freedom; DWLS, diagonal weighted least squares; HLQ, Health Literacy Questionnaire; RMSEA, root mean square error of approximation; SD, standard deviation; SRMR, standardized root mean square residual; TLI, Tucker–Lewis index; χ^2^, Chi-square.

^a^ Items are truncated. Full items are available from the authors.

^b^ Difficulty level for scales 1–5 was calculated as the proportion responding disagree and strongly disagree against agree or strongly agree.

Difficulty level for scales 6–9 was calculated as the proportion responding cannot do, very difficult, or quite difficult as against quite easy and very easy.

On the one hand, by items (44 items), “Discuss things with healthcare providers until you understand…” (Scale 6), “Decide which healthcare provider you need to see…” (Scale 7), “Work out what the best care is for you” (Scale 7), and “Read and understand all the information on medication labels” (Scale 9) were perceived as difficult (≥ 60%). On the other hand, “I have at least one person who can come to medical,” “When I feel ill, the people around me really understand,” and “I have strong support from family or friends” on Scale 4 were perceived as easy (≤ 10%) (Table 2).

### Psychometric properties of the Korean version of the HLQ

Table 2 shows the results of CFA performed to evaluate the construct validity of the HLQ. First, the fit of the nine one-factor CFA models was analyzed. Except for Scale 5 (CFI = 0.967, TLI = 0.935, SMSR = 0.061), the CFI and TLI were 0.95 or higher, and SMSR were below 0.08 (CFI: 0.987–0.998; TLI: 0.975–0.994; SRMR: 0.016–0.034). Moreover, RMSEA of scales 2 and 4 were 0.081 and 0.074, respectively, indicating an acceptable fit. However, the RMSEA of the remaining scales was 0.1 or higher. Factor loadings for each scale were satisfactory, at ≥ 0.60 (0.601–0.924) for all items.

Second, the fitness of a full nine-factor CFA model without correlated residuals or cross-loadings was analyzed, and the fit indices met the cutoffs (χ^2^ DWLS (df = 866) = 1993.353, *p* < 0.001, CFI = 0.975, TLI = 0.973, SRMR = 0.054, RMSEA = 0.056). Factor loading was satisfactory or better, at ≥ 0.60 (0.700–0.953) for 43 out of 44 items. The factor loading of the remaining item (“I have at least one person who can come to medical…”) was 0.570, and close to a satisfactory level.

Discriminant validity was determined by analyzing the inter-factor correlations among the nine scales (Table 3). The inter-factor correlations ranged from 0.373 (scales 3 and 4) to 0.897 (scales 6 and 7). Of 36 correlations, six were ≥ 0.80. The correlations ranged from 0.373–0.657 in Part 1 and 0.809–0.897 in Part 2 of HLQ. A high inter-factor correlation (≥ 0.80) suggests a potentially poor discriminant validity of the HLQ.

**Table 3:** Inter-factor correlations among the nine scales (n = 419).

**Scale**	**Part 1**					**Part 2**			
	**Scale 1**.	**Scale 2**.	**Scale 3**.	**Scale 4**.	**Scale 5**.	**Scale 6**.	**Scale 7**.	**Scale 8**.	**Scale 9**.
Scale 1.	1.000								
Scale 2.	0.640	1.000							
Scale 3.	0.603	0.657	1.000						
Scale 4.	0.439	0.407	0.373	1.000					
Scale 5.	0.592	0.710	0.698	0.457	1.000				
Scale 6.	0.517	0.540	0.406	0.433	0.501	1.000			
Scale 7.	0.484	0.565	0.404	0.468	0.483	0.897	1.000		
Scale 8.	0.422	0.585	0.406	0.413	0.502	0.809	0.891	1.000	
Scale 9.	0.438	0.554	0.415	0.388	0.509	0.814	0.860	0.884	1.000

Results of Pearson correlation analysis. Scale 1. Feeling understood and supported by healthcare providers (HPS); Scale 2. Having sufficient information to manage my health (HSI); Scale 3. Actively managing my health (AMH); Scale 4. Social support for health (SS); Scale 5. Appraisal of health information (CA); Scale 6. Ability to actively engage with healthcare providers (AE); Scale 7. Navigating the healthcare system (NHS); Scale 8. Ability to find good health information (HFI); Scale 9. Understand health information well enough to know what to do (UHI).

The CR scores of all nine scales of the HLQ were high at 0.8 or higher. The CR was the highest for Scale 7 (Navigating the healthcare system; 0.933) and lowest for Scale 4 (Social support for health; 0.806) (Table 2).

## Discussion

The HLQ is an instrument enabling a broad assessment of health literacy. It has been translated into multiple languages worldwide, currently including Korean, to measure health literacy in individuals or groups [15]. In the present study, the Korean translation of the HLQ was finalized, and psychometric validity evidence was presented. We applied a rigorous nine-factor confirmatory factor analysis (with no cross-loading or correlated residuals) and found that all items loaded strongly on their hypothesized factor. We also demonstrated that all scales had good to excellent reliability.

The Korean version of the HLQ appears to be more difficult for respondents to score higher than the original version [15] (Part 1: 10.0 ~ 38.0%, Part 2: 7.0~42.0%). However, the level of difficulty was similar to the Danish (Part 1: 45.8%, Part 2: 50.9%) [17] and French (Part 1: 45.1%, Part 2: 69.1%) [19]. The reason may be pertinent to the differences in the healthcare delivery system and cultural sensitivity in the country where the original instrument was developed. Part 2 was perceived as more difficult (53.1~55.7%) than Part 1 (11.6~46.7%), similar to the French version [19]. Part 1 consisted of questions about the subject’s health information search experience and utilization level. Part 2 consisted of questions about how difficult it was for participants to search for and use health information, contributing to the perceived difficulty. Among nine scales in the HLQ, Scale 7 (Navigating the healthcare system; 55.7%) and Scale 6 (Ability to actively engage with healthcare providers; 55.4%) were perceived as the most difficult. This suggests that individuals find “navigating the healthcare system” and “engaging with healthcare providers” challenging. Our study population predominantly comprised healthy individuals who may less frequently consult a healthcare provider about their health than those with a chronic condition. Thus, they may have indicated “cannot do or always difficult” regardless of the appropriateness of the items. Thus, it is necessary to evaluate HLQ levels and perceived difficulties exclusively among individuals with chronic diseases.

We performed CFA to test the construct validity of the HLQ. The one-factor CFA models for nine scales had a close fit overall, confirming unidimensionality. This result is similar to the CFA performed in the Danish [17] and French versions [19]. The factor loading was below 0.6 for between two and 13 items in the other versions of the HLQ, including the Danish [17] and French versions [19]. However, we found all items loaded onto a single factor (≥ 0.6) in our analysis. Thereafter, we fitted the data to the nine-factor CFA model, and the fit indices met the criteria, thus confirming a good model fit. This study is a better fit than that reported for the Danish [17] and French versions [19]. The Korean HLQ has satisfactory construct validity based on these fit indices and factor loadings.

All nine factors of the Korean version of the HLQ had a CR of 0.80 or higher. Similarly, a high CR was reported for the original [15], Danish [17], and French versions [19]. Therefore, HLQ has good reliability. Discriminant validity was evaluated using inter-factor correlations. Scales 1–5 demonstrated good discriminant validity. However, discriminate validity was less clear for Scales 6–9, with the correlation coefficient > 0.80. The English [15] and German versions [18] reported similar results. The construct definitions and the item content indicate that the constructs are measuring different elements of health literacy. However, the high correlations among these scales may be attributed to addressing a common overarching issue related to ability or confidence in finding (Scale 8), understanding (Scale 9), navigating (Scale 7), and engaging (Scale 6). This suggests that there may be a high-order factor present [15], or the factors are causally related [16]. Future studies should investigate this issue in diverse settings among people with a wide range of health literacy strengths and challenges.

The findings of this study indicate that the Korean version of the HLQ is likely to be a useful tool for understanding health literacy in healthy individuals and people with health conditions in Korea. It may also be useful for researching adherence to health education and self-management interventions and exploring determinants of health-related quality of life.

This study has a few limitations. First, adults aged 19 years and older were convenience-sampled in the Seoul Metropolitan area in Korea. More than half of the study participants were highly educated, and the study was conducted in an urban area. Thus, generalizing the results to the general population requires caution, and replication studies on the general population from more diverse communities are needed. Second, we collected data using a QR code; thus, individuals with easy access to electronics and the Internet may have been primarily recruited. Hence, subsequent studies should consider participants’ access to health-related information and collect data using various media types. The Korean version of the HLQ was developed through a systematic translation process using forward and carefully guided by detailed item intents to ensure good linkage to the originally-intended constructs developed in Australia. However, the instrument is still vulnerable to systematic errors due to cultural gaps, such as differences in the healthcare delivery systems between the two countries. Therefore, caution should be applied when interpreting results.

## Conclusion

Health literacy allows individuals to engage in daily health practices; hence, it is considered essential to meet health needs. Health literacy varies across individuals; therefore, healthcare providers should assess patients’ comprehensive health literacy before providing treatment, nursing care, or tests. The present study translated the HLQ into Korean and tested this version in an urban population. The validity and reliability results support the validity of data from the Korean version of the HLQ in the study context. These initial findings, i.e., strong psychometric properties, indicate that the Korea version of the HLQ is a useful tool to generate an in-depth evaluation of patients’ experiences, skills, strengths, and needs pertaining to health literacy.

## Supporting information

S1 Checklist (DOC)
